# Variable density of CD8+ and CD25+ programmed cell death-1 (PD-1) ligand (PD-L1) CD4+ T cells within the tumor microenvironment causes differential responses to the PD-1/PD-L1 blockade

**DOI:** 10.1186/2051-1426-3-S2-P423

**Published:** 2015-11-04

**Authors:** Si-Pei Wu, Yi-Long Wu

**Affiliations:** 1 Guangdong General Hospital, Guangzhou, People's Republic China

## 

The programmed cell death-1 (PD-1)/programmed cell death-1 ligand (PD-L1) pathway has been shown to play a pivotal role in tumor evasion. Inhibition of PD-1 and its ligand PD-L1 using an immune checkpoint inhibitor has emerged as a promising immunotherapy for the treatment of various types of cancer. The expression of PD-L1 in tumor cells and tumor-infiltrating lymphocytes (TILs) has been shown to be correlated with improved efficacy of antibodies against PD-1 or PD-L1. Moreover, the density of CD8^+^ TILs has been shown to be correlated with the response to immunotherapy. In this study, we examined the expression of PD-L1, CD25, PD-1, FoxP3, CD4, CD8, and EpCAM in 42 peripheral blood samples and fresh tumor specimens using multiparametric flow cytometry. Our results showed that the percentages of PD-L1-expressing CD25^+^ CD4^+^ T cells were significantly higher in TILs than in peripheral blood lymphocytes (PBLs; TILs: mean, 48.6%; range, 23.6–81.5% versus PBLs: mean, 35.4%; range, 16.8–64.3%; *P* < 0.001). The density of CD25^+^ PD-L1^+^ CD4^+^ TILs positively correlated with that of PD-1^+^ CD8^+^ TILs but negatively correlated with interferon (IFN)-γ^+^ and tumor necrosis factor (TNF)-β^+^ CD8^+^ TILs. However, the high ratio of CD8^+^ TILs to that of CD25^+^ PD-L1^+^ CD4^+^ TILs or to EpCAM^+^ tumor cells were associated with high percentages of IFN-γ^+^ and TNF-β^+^ CD8^+^ TILs. Moreover, inhibition of PD-L1 and PD-1 decreased the density of CD25^+^ PD-L1^+^ CD4^+^ cells and PD-1^+^ CD8^+^ TILs but increased the percentage of IFN-γ^+^ and TNF-β^+^ CD8^+^ cells. High ratios of CD8^+^ TILs to CD25^+^ PD-L1^+^ CD4^+^ TILs or to EpCAM^+^ tumor cells enhanced the activity of tumor-specific CD8^+^ T cells after PD-1/PD-L1 blockade therapy. Taken together, our results highlighted the importance of CD25^+^ PD-L1^+^ CD4^+^ TILs in mediating the tumor microenvironment immune response. Our findings also indicated that high ratios of CD8^+^ TILs to CD25^+^ PD-L1^+^ CD4^+^ TILs or to EpCAM^+^ tumor cells in patients may be more effective after PD-1/PD-L1 blockade therapy. Thus, the variable density of CD8^+^ and CD25^+^ PD-L1^+^ CD4^+^ T cells within the tumor microenvironment caused differential responses to PD-1/PD-L1 blockade.

**Figure 1:**
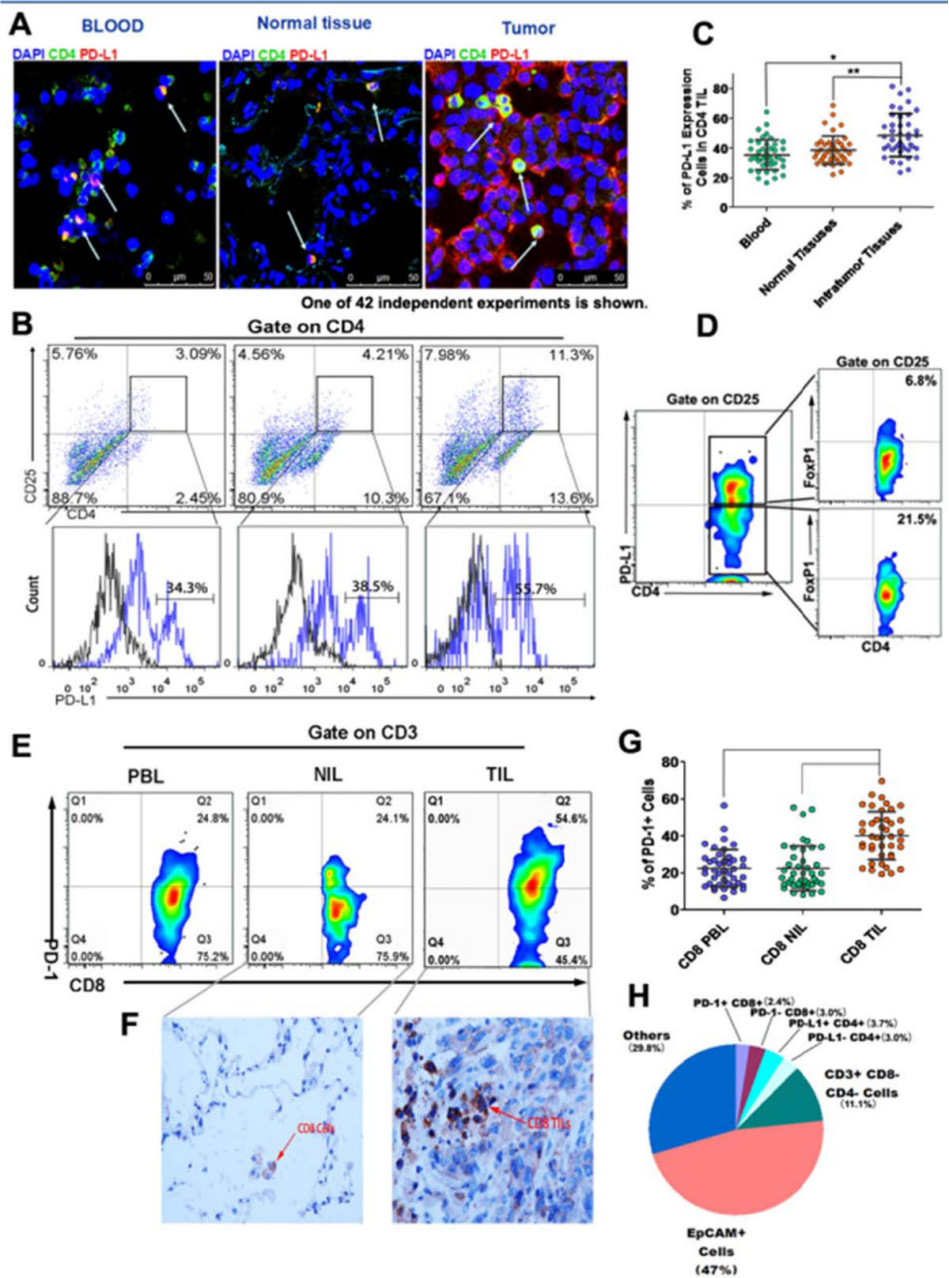


**Figure 2:**
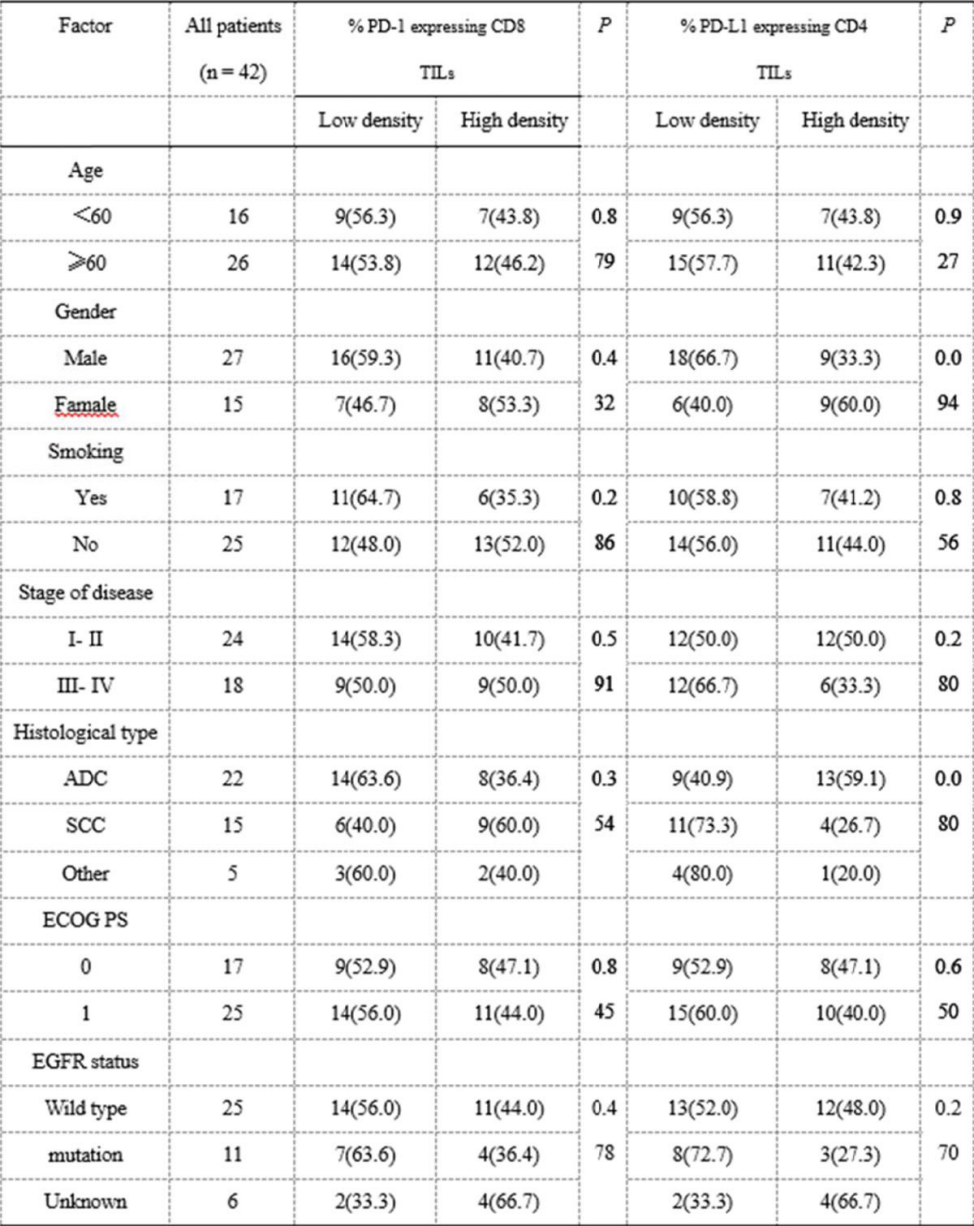
Correlation between the clinicopathologic characteristics and PD-1+CD8 TIL or PD-L1+CD4 TIL in 42 lung cancer patients.

